# Childhood experiences of parenting and cancer risk at older ages: findings from the English Longitudinal Study of Ageing (ELSA)

**DOI:** 10.1007/s00038-018-1117-3

**Published:** 2018-06-14

**Authors:** Panayotes Demakakos, Georgios P. Chrousos, Jane P. Biddulph

**Affiliations:** 1 Department of Epidemiology and Public Health, University College London, 1-19 Torrington Place, London, WC1E 6BT UK; 2 First Department of Pediatrics, National and Kapodistrian University of Athens Medical School, Aghia Sophia Children’s Hospital, Athens, Greece

**Keywords:** Parenting, Cancer, Stress, Life course, Aging, Childhood, Cohort

## Abstract

**Objectives:**

Despite the importance of childhood experiences for adult health and psychosocial factors for cancer development, parenting, a key childhood psychosocial exposure, has yet to be studied in relation to cancer risk at older ages. We examined whether childhood experiences of poor-quality parenting are associated with an increased risk of cancer at older ages.

**Methods:**

We used a sample of 4471 community dwellers aged ≥ 55 years in 2007. Poor-quality parenting was defined as low levels of parental care and high levels of parental overprotection.

**Results:**

Overall poorer experiences of parenting, decreasing parental care and increasing parental overprotection were associated with increased risk of incident all-site and skin cancer in men, but not in women. Increasing paternal overprotection was also associated with increased risk of incident colorectal cancer in men. Overall poorer experiences of parenting and increasing paternal overprotection were associated with increased risk of prevalent all-site and colorectal cancer in women. Adjustment for covariates explained a small part of these associations.

**Conclusions:**

Older adults who reported childhood experiences of poorer quality parenting appear to have an increased risk of cancer. These findings improve our understanding of the role of psychosocial factors in cancer over the life course.

**Electronic supplementary material:**

The online version of this article (10.1007/s00038-018-1117-3) contains supplementary material, which is available to authorized users.

## Introduction

A substantial body of literature highlights the importance of early life and childhood experiences for later life health. Evidence also suggests that psychosocial factors are strongly associated with cancer incidence and mortality (Chida et al. 2008), yet research on the association between childhood psychosocial experiences and cancer development and progression in adulthood remains limited. The literature has mostly focused on financial hardship and parental socio-economic position (SEP) and cancer mortality (Galobardes et al. 2008), with some additional research on the association between adverse childhood experiences, such as abuse and neglect, and cancer development in adulthood (Brown et al. 2010, 2013; Norman et al. 2012; Kelly-Irving et al. 2013a; Hyland et al. 2013). Parenting and parent–child relationships, despite their pivotal role in childhood, have rarely been studied with regard to the risk of developing and dying from cancer in adulthood (Shaffer et al. 1982; Demakakos et al. 2016b). To our knowledge, no study has examined the association between childhood experiences of poor-quality parenting and cancer development at older ages, where most cancer cases occur.

Our conceptualization of parenting focuses on the bond between the parent and child and security of attachment as a foundation of human development (Parker et al. 1979; Parker 1983). It draws on existing typologies of parenting, which on the basis of a synthesis of different scientific traditions focus on two fundamental dimensions of parenting: parental control/demandingness and warmth/responsiveness and their interrelationships (Baumrind 1991; Darling and Steinberg 1993; Simons and Conger 2007; Oliver et al. 2014). Based on these foundations, we measured childhood experiences of parenting on the basis of how much care and affection our participants received by their parents and how overprotective their parents were. We defined poor parenting as low levels or lack of parental care and affection and high levels of overprotection.

We hypothesize that poor-quality parenting is a pluripotent and omnipresent childhood stressor and as such might be associated with cancer risk at older ages. This hypothesis is consistent with a proposed model of childhood adversity and cancer in adulthood, which suggested that familial factors can cause acute and chronic stress responses, which can result in increased cancer risk in adulthood directly via neuroendocrine, immune and epigenetic changes or indirectly via behavioural adaptations (Kelly-Irving et al. 2013b). Existing approaches on childhood stress and its impact on adult health can be used to gain a better understanding of the biological mechanisms of the hypothesized association. The main tenets of these approaches are two: (a) the chronic stress-related alterations in the function of the neuroendocrine and immune systems and their subsequent disregulation and (b) the deleterious effect of chronic stress on the developing brain (Gunnar and Quevedo 2007; Lupien et al. 2009; Nusslock and Miller 2016).

Based on these assumptions, we examined whether participants with experiences of poorer quality parenting in childhood had an increased risk of incident cancer in a national sample of men and women aged ≥ 55 years. We also investigated whether experiences of poorer quality parenting in childhood were associated with lifetime prevalence of cancer. Our investigation focused on all-site cancer and cancer sites, for which we have data. The all-site cancer analyses should provide answers to our main question about whether poor-quality parenting is associated with cancer development at older ages. The site-specific analyses can provide additional information on specific carcinogenic pathways and substantially add to our understanding of the examined associations. Our study is timely, adds evidence to the current paucity of literature and can improve our understanding of the role of psychosocial factors in cancer over the life course. Because there are significant sex differences in the aetiology and epidemiology of cancer as well as the distribution of potential mediators such as behavioural and psychosocial factors and boys and girls may respond differently to different parenting styles, we stratified our analyses according to sex.

## Methods

### Participants

Data came from the English Longitudinal Study of Ageing (ELSA), which is a prospective observational study of community-dwelling people aged ≥ 50 years that was designed to be nationally representative. The ELSA baseline interview was in 2002–2003. Follow-up interviews took place at regular two-year intervals after the baseline. We used data from the life history add-on survey, which took place in 2007 and collected retrospective information about the experiences and life circumstances of the ELSA participants from birth to the age they joined ELSA, and the second follow-up ELSA interview in 2006–2007. The cancer data come from six ELSA interviews stretching from 2002–2003 to 2012–2013.

Of the 11,391 individuals who were present at baseline in 2002–2003, 6199 participated in the life history survey, of which 4471 constituted the sample from which two analytical samples were selected after the exclusion of those who did not complete the childhood experiences questionnaire (*n* = 931); did not report on their childhood experiences of parenting (*n* = 483); or were not reared by both natural parents (*n* = 314). The all-site cancer incidence analytical sample comprised 3634 participants after excluding participants with no follow-up data (*n* = 302); prevalent cases of cancer (*n* = 422); and those with missing values in variables used in the analyses (*n* = 113). The skin, colorectal and breast cancer incidence analyses samples comprised slightly more participants as in these analyses we only excluded prevalent cases of a specific cancer site and not all prevalent cases of cancer. The sample that was used in all prevalence analyses comprised 4361 participants after the exclusion of participants with missing values in variables used in the analyses (*n* = 110). ELSA has been approved by the National Research Ethics Service, and informed consent has been obtained by the participants. A detailed description of ELSA can be found at: http://www.elsa-project.ac.uk/.

### Assessment of childhood experiences of parenting

Parenting was measured using the seven-item Parental Bonding Instrument (PBI) (Parker et al. 1979; Todd et al. 1994), which was part of the ELSA life history survey. The PBI is designed to be used by adults and retrospectively measures their experiences of their parents’ parenting style at age ≤ 15 years. PBI focuses on two fundamental dimensions of parenting, care and overprotection. Parental care refers to parental behaviours reflecting emotional warmth, affection, empathy, closeness and care for one’s child as opposed to emotional coldness, indifference and neglect (Parker et al. 1979). Parental overprotection refers to parental behaviours reflecting control, overprotection, intrusion, excessive contact and prevention of independent behaviour as opposed to allowance of independence and autonomy (Parker et al. 1979). The seven-item PBI includes three care and four overprotection items. According to the PBI scoring system, optimal parenting is characterized by high levels of care and low levels of overprotection. We derived care and overprotection scores for both natural parents and a parenting summary score. To avoid the unnecessary exclusion of participants with few missing values (*n* = 93 and *n* = 182 in the prevalence analyses and *n* = 67 and *n* = 146 in the incidence analyses for men and women, respectively), we substituted up to one missing value per subscale with the mean score of that subscale. As the analyses of both imputed and complete data produced similar results, we used as a basis for our work the former and for comparison reasons present the latter in eTable 1.

### Cancer

We used self-reported doctor diagnosis of cancer (excluding minor skin cancer, which had not been measured in ELSA) and date of diagnosis to ascertain both prevalent and incident cases. Participants who reported being diagnosed with cancer at baseline (that is the date of their life history interview in 2007) or earlier were characterised as prevalent cases. Participants who had not been diagnosed with cancer before and reported being diagnosed after the baseline were characterised as incident cases. The self-reported site of the primary cancer was available for a limited range of common sites, and for most of these sites, the number of observed cases was small. Nevertheless, we were able to perform analyses of the associations between childhood experiences of parenting and prevalent and incident colorectal, skin and breast cancer. Secondary cancers (metastases), which chronologically followed the onset of a primary cancer, were not included in the analyses.

### Covariates

We measured age, childhood and adult SEP, participant marital and parenthood statuses, elevated depressive symptoms, positive affect, body mass index, waist circumference, smoking, physical activity, memory, social support and number of problems with social relationships. The rationale for choosing these covariates and a more detailed description of them can be found elsewhere (Demakakos et al. 2016b).

### Statistical analysis

For the incidence analyses, once we established that the proportionality assumption held, we estimated sex-specific Cox proportional hazard regression models of the associations between the parenting measures and incident cancer. Due to a very small number of cases (*n* = 6), we did not examine the association between parenting experiences and incident colorectal cancer in women. Survival time (in months) was the time that elapsed from the date of the baseline interview in 2007 to the first of either the date of diagnosis or censoring (that is the date of the last ELSA interview a participant had). For the prevalence analyses, we estimated logistic regression models of the associations between the parenting measures and prevalent cancer.

Models were initially adjusted for age, then for childhood SEP and finally for all other covariates. In supplementary analyses, we additionally adjusted our models for self-reported baseline comorbidities, adverse childhood experiences and childhood health problems that are known to be associated with parenting, such as epilepsy, asthma, diabetes, hearing problems and limiting disability (Pinquart 2013).

## Results

Men and women who reported having poorer quality parenting experiences in childhood were more likely to report that they lived in a household with fewer books at age 10 years, lower levels of positive affect, more depressive symptoms and problems with social relationships, and less social support than those who reported being raised with a better parenting style. In addition, women with experiences of poorer quality parenting in childhood were more likely to be ex-smokers (see eTable 2).

### Cancer incidence

We observed 112 cases of incident cancer in men over a median follow-up of 5.3 years (mean: 4.7 years) and 85 cases in women over a median follow-up of 5.3 years (mean: 4.8 years). Experiences of poorer quality parenting in childhood were associated with increased risk of incident all-site cancer in men, but not in women (Table 1). Paternal overprotection and decreasing paternal care were associated with increased risk of incident all-site cancer in men; the former more strongly than the latter. Maternal overprotection and decreasing maternal care were also associated with increased risk of incident all-site cancer, but these associations were slightly weaker. Adjustment for covariates (Table 1) and additional adjustments for baseline chronic diseases, adverse childhood experiences and childhood health problems explained a relatively small part of these associations (eTable 3). Analyses by cancer site suggested that all parenting measures were associated with the risk of incident skin cancer in men (Table 2), while paternal overprotection was also strongly associated with the risk of incident colorectal cancer in men (Table 2).

**Table 1:** Associations between all-site cancer incidence and prevalence and parenting measures by sex, English Longitudinal Study of Ageing, 2007–2013

	Cancer incidence		Cancer prevalence	
	Men	Women	Men	Women
No. of participants	1650	1984	1986	2375
No. of cases	112	85	194	262
Person years of follow-up	7712	9485	n/a	n/a

	Hazard ratio (95% CI)^a^		Odds ratio (95% CI)^b^	
Parenting summary score(both parents)—reversed (range 0—highest quality parenting to 42—lowest quality parenting)				
Model 1^c^	1.05 (1.02 to 1.08)	1.00 (0.97 to 1.04)	0.99 (0.96 to 1.02)	1.02 (1.00 to 1.04)
Model 2^d^	1.05 (1.02 to 1.09)	1.00 (0.97 to 1.04)	1.00 (0.97 to 1.03)	1.02 (1.00 to 1.04)
Model 3^e^	1.05 (1.02 to 1.09)	1.00 (0.96 to 1.04)	1.00 (0.97 to 1.03)	1.02 (1.00 to 1.04)
Model 4^f^	1.05 (1.01 to 1.08)	1.00 (0.96 to 1.04)	1.00 (0.97 to 1.03)	1.02 (1.00 to 1.04)
Maternal Care score—reversed (range 0—highest levels of care to 9—lowest levels of care)				
Model 1^c^	1.10 (0.99 to 1.22)	1.00 (0.90 to 1.11)	0.98 (0.89 to 1.07)	1.00 (0.94 to 1.07)
Model 2^d^	1.12 (1.01 to 1.24)	1.01 (0.90 to 1.12)	1.00 (0.91 to 1.10)	1.01 (0.95 to 1.08)
Model 3^e^	1.12 (1.01 to 1.24)	0.99 (0.89 to 1.11)	1.00 (0.91 to 1.10)	1.02 (0.95 to 1.08)
Model 4^f^	1.10 (0.99 to 1.23)	1.00 (0.89 to 1.12)	1.00 (0.91 to 1.10)	1.01 (0.94 to 1.08)
Maternal overprotection score (range 0—lowest levels of overprotection to 12—highest levels of overprotection)				
Model 1^c^	1.09 (0.99 to 1.20)	0.99 (0.89 to 1.10)	0.99 (0.91 to 1.07)	1.06 (0.99 to 1.12)
Model 2^d^	1.10 (1.00 to 1.21)	0.99 (0.89 to 1.10)	0.99 (0.92 to 1.08)	1.06 (1.00 to 1.13)
Model 3^e^	1.10 (1.00 to 1.21)	0.98 (0.88 to 1.09)	0.99 (0.91 to 1.08)	1.06 (1.00 to 1.13)
Model 4^f^	1.08 (0.98 to 1.19)	0.98 (0.88 to 1.10)	1.00 (0.92 to 1.08)	1.06 (1.00 to 1.13)
Paternal care score—reversed (range 0—highest levels of care to 9—lowest levels of care)				
Model 1^c^	1.13 (1.03 to 1.24)	1.03 (0.92 to 1.15)	0.94 (0.86 to 1.02)	1.03 (0.96 to 1.10)
Model 2^d^	1.15 (1.05 to 1.26)	1.04 (0.93 to 1.16)	0.97 (0.89 to 1.06)	1.03 (0.96 to 1.11)
Model 3^e^	1.15 (1.05 to 1.26)	1.03 (0.92 to 1.15)	0.97 (0.89 to 1.06)	1.04 (0.97 to 1.11)
Model 4^f^	1.14 (1.03 to 1.25)	1.03 (0.92 to 1.17)	0.97 (0.88 to 1.06)	1.03 (0.96 to 1.11)
Paternal overprotection score (range 0—lowest levels of overprotection to 12—highest levels of overprotection)				
Model 1^c^	1.16 (1.05 to 1.28)	0.99 (0.89 to 1.11)	1.01 (0.93 to 1.10)	1.08 (1.01 to 1.15)
Model 2^d^	1.17 (1.06 to 1.29)	1.00 (0.89 to 1.11)	1.02 (0.93 to 1.11)	1.09 (1.02 to 1.16)
Model 3^e^	1.17 (1.06 to 1.29)	0.99 (0.88 to 1.10)	1.02 (0.93 to 1.11)	1.09 (1.02 to 1.17)
Model 4^f^	1.15 (1.03 to 1.28)	0.99 (0.88 to 1.11)	1.03 (0.95 to 1.13)	1.09 (1.02 to 1.17)

^a^Hazard ratios denote hazard change per unit change in the predictor

^b^Odds ratios denote change in the odds per unit change in the predictor

^c^This is the unadjusted association

^d^Model 2 is adjusted for age

^e^As model 2, plus adjustment for childhood socio-economic position (i.e. ownership of the first ever permanent residence, number of books in the household at age 10 years and father’s or main carer’s occupational class at age 14 years)

^f^As model 3, plus adjustment for adult socio-economic position (i.e. education and total net household wealth), marital status, parenthood status, obesity (i.e. body mass index and waist circumference), memory, unhealthy behaviours (i.e. smoking and physical activity), social factors (i.e. social support and number of problems with social relationships), elevated depressive symptoms and positive affect

**Table 2:** Associations between site-specific cancer incidence and parenting measures by sex, English Longitudinal Study of Ageing, 2007–2013

	Men		Women	
	Skin	Colorectal	Skin	Breast
No. of participants	1799	1801	2207	2108
No. of cases	15	13	17	30
Person years of follow-up	8588	8625	10,733	10,199
Parenting summary score (both parents)—reversed (range 0—highest quality parenting to 42—lowest quality parenting)				
Model 1 HR (95% CI)^a^	1.17 (1.08 to 1.26)	1.07 (0.97 to 1.17)	1.00 (0.93 to 1.08)	1.02 (0.96 to 1.08)
Model 2 HR (95% CI)^b^	1.17 (1.08 to 1.26)	1.07 (0.97 to 1.17)	1.01 (0.93 to 1.10)	1.01 (0.97 to 1.07)
Model 3 HR (95% CI)^c^	1.15 (1.06 to 1.24)	1.07 (0.98 to 1.17)	1.01 (0.93 to 1.10)	1.01 (0.95 to 1.07)
Model 4 HR (95% CI)^d^	1.20 (1.06 to 1.34)	1.14 (1.02 to 1.28)	0.99 (0.90 to 1.08)	1.02 (0.96 to 1.08)
Maternal care score—reversed (range 0—highest levels of care to 9—lowest levels of care)				
Model 1 HR (95% CI)^a^	1.36 (1.08 to 1.72)	1.12 (0.83 to 1.51)	1.03 (0.82 to 1.30)	0.94 (0.78 to 1.14)
Model 2 HR (95% CI)^b^	1.38 (1.10 to 1.74)	1.12 (0.82 to 1.52)	1.06 (0.79 to 1.32)	0.93 (0.77 to 1.13)
Model 3 HR (95% CI)^c^	1.29 (1.04 to 1.61)	1.15 (0.85 to 1.54)	1.06 (0.78 to 1.35)	0.92 (0.76 to 1.12)
Model 4 HR (95% CI)^d^	1.33 (1.02 to 1.74)	1.24 (0.90 to 1.72)	0.99 (0.71 to 1.25)	0.93 (0.75 to 1.14)
Maternal overprotection score (range 0—lowest levels of overprotection to 12—highest levels of overprotection)				
Model 1 HR (95% CI)^a^	1.40 (1.12 to 1.75)	1.10 (0.83 to 1.45)	1.00 (0.79 to 1.26)	1.04 (0.87 to 1.24)
Model 2 HR (95% CI)^b^	1.41 (1.14 to 1.76)	1.10 (0.83 to 1.45)	1.01 (0.79 to 1.29)	1.03 (0.87 to 1.23)
Model 3 HR (95% CI)^c^	1.38 (1.10 to 1.73)	1.12 (0.86 to 1.46)	1.02 (0.80 to 1.30)	1.03 (0.87 to 1.23)
Model 4 HR (95% CI)^d^	1.49 (1.12 to 2.00)	1.24 (0.90 to 1.72)	0.99 (0.76 to 1.28)	1.05 (0.88 to 1.26)
Paternal care score—reversed (range 0—highest levels of care to 9—lowest levels of care)				
Model 1 HR (95% CI)^a^	1.34 (1.07 to 1.68)	1.04 (0.79 to 1.39)	1.05 (0.82 to 1.34)	1.05 (0.87 to 1.26)
Model 2 HR (95% CI)^b^	1.38 (1.10 to 1.73)	1.05 (0.79 to 1.39)	1.08 (0.84 to 1.39)	1.03 (0.86 to 1.25)
Model 3 HR (95% CI)^c^	1.30 (1.03 to 1.65)	1.07 (0.80 to 1.42)	1.07 (0.83 to 1.38)	1.03 (0.85 to 1.25)
Model 4 HR (95% CI)^d^	1.38 (1.02 to 1.86)	1.19 (0.85 to 1.65)	0.97 (0. 73 to 1.30)	1.03 (0.84 to 1.26)
Paternal overprotection score (range 0—lowest levels of overprotection to 12—highest levels of overprotection)				
Model 1 HR (95% CI)^a^	1.51 (1.21 to 1.89)	1.40 (1.08 to 1.81)	0.94 (0.73 to 1.20)	1.13 (0.95 to 1.34)
Model 2 HR (95% CI)^b^	1.52 (1.22 to 1.90)	1.40 (1.08 to 1.81)	0.95 (0.74 to 1.23)	1.12 (0.94 to 1.33)
Model 3 HR (95% CI)^c^	1.60 (1.25 to 2.05)	1.39 (1.09 to 1.78)	0.95 (0.74 to 1.22)	1.11 (0.94 to 1.32)
Model 4 HR (95% CI)^d^	2.08 (1.41 to 3.07)	1.64 (1.21 to 2.24)	0.92 (0.70 to 1.20)	1.15 (0.96 to 1.37)

*HR* Hazard Ratio

Hazard ratios denote hazard change per unit change in the predictor

^a^This is the unadjusted association

^b^Model 2 is adjusted for age

^c^As model 2, plus adjustment for childhood socio-economic position (i.e. ownership of the first ever permanent residence, number of books in the household at age 10 years and father’s or main carer’s occupational class at age 14 years)

^d^As model 3, plus adjustment for adult socio-economic position (i.e. education and total net household wealth), marital status, parenthood status, obesity (i.e. body mass index and waist circumference), memory, unhealthy behaviours (i.e. smoking and physical activity), social factors (i.e. social support and number of problems with social relationships), elevated depressive symptoms and positive affect

### Cancer prevalence

We identified 194 and 262 cases of prevalent cancer in men and women, respectively. The associations between the parenting measures and cancer prevalence were weaker than the incidence-related associations and followed a different pattern, with experiences of poorer quality parenting in childhood being associated with increased risk of prevalent all-site cancer in women, but not in men (Table 1). Paternal overprotection was more strongly associated with all-site cancer prevalence than maternal overprotection. Adjustment for covariates did not affect these associations (Table 1 and eTable 4). Analyses of cancer sites indicated that there were strong associations between paternal and maternal overprotection scores, as well as the parenting summary score, and the risk of prevalent colorectal cancer in women (Table 3). We also found a weak positive association between maternal care and the risk of prevalent breast cancer, with decreasing maternal care being associated with decreased risk of prevalent breast cancer (Table 3). Further, we found a borderline positive association between paternal overprotection and the risk of prevalent skin cancer in men (Table 3).

**Table 3:** Associations between site-specific cancer prevalence and parenting measures by sex, English longitudinal study of ageing, 2007

	Men		Women		
	Skin	Colorectal	Skin	Colorectal	Breast
No. of participants	1986	1986	2375	2375	2375
No. of cases	36	32	23	28	123
Parenting summary score (both parents)—reversed (range 0—highest quality parenting to 42—lowest quality parenting)					
Model 1 OR (95% CI)^a^	1.03 (0.97 to 1.09)	0.96 (0.90 to 1.02)	0.98 (0.92 to 1.05)	1.09 (1.03 to 1.15)	0.99 (0.96 to 1.02)
Model 2 OR (95% CI)^b^	1.03 (0.98 to 1.10)	0.96 (0.90 to 1.03)	0.99 (0.92 to 1.06)	1.10 (1.04 to 1.17)	0.99 (0.96 to 1.02)
Model 3 OR (95% CI)^c^	1.03 (0.97 to 1.10)	0.96 (0.90 to 1.03)	0.99 (0.92 to 1.06)	1.10 (1.04 to 1.17)	0.99 (0.96 to 1.02)
Model 4 OR (95% CI)^d^	1.05 (0.98 to 1.12)	0.96 (0.89 to 1.03)	0.98 (0.91 to 1.06)	1.11 (1.04 to 1.18)	1.00 (0.96 to 1.03)
Maternal Care score—reversed (range 0—highest levels of care to 9—lowest levels of care)					
Model 1 OR (95% CI)^a^	1.07 (0.89 to 1.29)	0.98 (0.78 to 1.22)	0.90 (0.71 to 1.13)	1.09 (0.92 to 1.30)	0.87 (0.78 to 0.96)
Model 2 OR (95% CI)^b^	1.09 (0.90 to 1.32)	1.00 (0.78 to 1.24)	0.91 (0.71 to 1.15)	1.11 (0.94 to 1.32)	0.86 (0.78 to 0.96)
Model 3 OR (95% CI)^c^	1.09 (0.90 to 1.32)	0.99 (0.79 to 1.23)	0.91 (0.72 to 1.16)	1.11 (0.93 to 1.32)	0.87 (0.78 to 0.97)
Model 4 OR (95% CI)^d^	1.12 (0.92 to 1.36)	0.99 (0.77 to 1.26)	0.91 (0.70 to 1.17)	1.10 (0.91 to 1.33)	0.87 (0.78 to 0.98)
Maternal overprotection score (range 0—lowest levels of overprotection to 12—highest levels of overprotection)					
Model 1 OR (95% CI)^a^	1.07 (0.90 to 1.27)	0.88 (0.73 to 1.08)	1.04 (0.85 to 1.27)	1.32 (1.12 to 1.56)	0.97 (0.89 to 1.07)
Model 2 OR (95% CI)^b^	1.08 (0.91 to 1.28)	0.89 (0.73 to 1.08)	1.05 (0.86 to 1.29)	1.36 (1.15 to 1.61)	0.97 (0.89 to 1.06)
Model 3 OR (95% CI)^c^	1.06 (0.89 to 1.27)	0.88 (0.72 to 1.08)	1.05 (0.86 to 1.30)	1.37 (1.15 to 1.62)	0.98 (0.89 to 1.07)
Model 4 OR (95% CI)^d^	1.09 (0.91 to 1.32)	0.87 (0.70 to 1.08)	1.03 (0.84 to 1.28)	1.39 (1.15 to 1.68)	0.99 (0.90 to 1.09)
Paternal Care score—reversed (range 0—highest levels of care to 9—lowest levels of care)					
Model 1 OR (95% CI)^a^	0.98 (0.82 to 1.18)	0.87 (0.70 to 1.07)	0.94 (0.74 to 1.19)	1.10 (0.91 to 1.32)	1.00 (0.91 to 1.11)
Model 2 OR (95% CI)^b^	1.02 (0.85 to 1.22)	0.88 (0.71 to 1.09)	0.95 (0.75 to 1.21)	1.12 (0.93 to 1.36)	1.00 (0.90 to 1.10)
Model 3 OR (95% CI)^c^	1.01 (0.84 to 1.21)	0.88 (0.71 to 1.08)	0.94 (0.74 to 1.20)	1.13 (0.93 to 1.37)	1.01 (0.91 to 1.11)
Model 4 OR (95% CI)^d^	1.03 (0.85 to 1.25)	0.87 (0.69 to 1.10)	0.92 (0.72 to 1.18)	1.14 (0.92 to 1.42)	1.03 (0.93 to 1.15)
Paternal overprotection score (range 0—lowest levels of overprotection to 12—highest levels of overprotection)					
Model 1 OR (95% CI)^a^	1.15 (0.96 to 1.38)	0.89 (0.72 to 1.10)	0.95 (0.76 to 1.18)	1.38 (1.18 to 1.62)	1.03 (0.94 to 1.13)
Model 2 OR (95% CI)^b^	1.16 (0.97 to 1.39)	0.90 (0.73 to 1.10)	0.96 (0.77 to 1.19)	1.42 (1.21 to 1.66)	1.03 (0.94 to 1.13)
Model 3 OR (95% CI)^c^	1.16 (0.97 to 1.39)	0.88 (0.71 to 1.09)	0.95 (0.76 to 1.18)	1.42 (1.21 to 1.68)	1.04 (0.95 to 1.14)
Model 4 OR (95% CI)^d^	1.21 (1.00 to 1.46)	0.87 (0.70 to 1.09)	0.94 (0.75 to 1.19)	1.49 (1.24 to 1.79)	1.05 (0.96 to 1.16)

*OR* Odds Ratio

Odds ratios denote change in the odds per unit change in the predictor

^a^This is the unadjusted association

^b^Model 2 is adjusted for age

^c^As model 2, plus adjustment for childhood socio-economic position (i.e. ownership of the first ever permanent residence, number of books in the household at age 10 years and father’s or main carer’s occupational class at age 14 years)

^d^As model 3, plus adjustment for adult socio-economic position (i.e. education and total net household wealth), marital status, parenthood status, obesity (i.e. body mass index and waist circumference), memory, unhealthy behaviours (i.e. smoking and physical activity), social factors (i.e. social support and number of problems with social relationships), elevated depressive symptoms and positive affect

## Discussion

Our findings suggest that experiences of poorer quality parenting in childhood are associated with cancer development in a national sample of community-dwelling men and women aged ≥ 55 years. We found that men with childhood experiences of poorer quality parenting were at increased risk of incident cancer. The father–son relationship appears to be an important childhood determinant of cancer incidence among older male offspring, with increasing paternal overprotection being more strongly associated with the risk of developing cancer than decreasing paternal care. The mother–son relationship also appears to be relevant, but its association with incident cancer was somewhat weaker. In women, an overall poorer quality parenting style and maternal and paternal overprotection were associated with cancer prevalence. As with cancer incidence in men, paternal overprotection was more strongly associated with increased risk of prevalent cancer than any other parenting dimension. Our findings taken together suggest that men and women whose parents’ parenting style was suboptimal and characterized by overprotection or limited amounts of care and affection may be at increased risk of developing cancer at older ages.

Research on the association between childhood experiences of parenting and cancer development in late adulthood is very limited. A study of 913 US middle-aged male physicians found that poor-quality father–son relationship, but not mother–son relationship, was associated with an increased risk of incident cancer (Shaffer et al. 1982), while a recent study found a negative dose–response association between parenting quality and cancer mortality risk in people aged 65–79 years (Demakakos et al. 2016b). Evidence also suggests an association between childhood experiences of abuse and neglect and an increased risk of cancer in early and mid-adulthood (Brown et al. 2010, 2013; Norman et al. 2012; Morton et al. 2012; Kelly-Irving et al. 2013a; Hyland et al. 2013).

Our study has strengths and weaknesses that need to be acknowledged. An obvious strength is the use of a national sample, which makes our findings more generalizable to the community-dwelling population aged ≥ 55 years. It is also important that we were able to adjust our models for depressive symptoms, positive affect and memory impairment and, thus, account for mood- and memory-related measurement biases. Further, in supplementary analyses, we provided evidence that poorer quality parenting was associated with increased cancer risk independently of other known childhood risk factors such as adverse childhood experiences and childhood diseases. Finally, the use of PBI, which is an instrument that is widely used to measure parenting experiences in adults, makes the replication of our findings easier.

Like all observational cohort studies, our study is potentially vulnerable to bias stemming from unaccounted confounders. It should be noted that the observed associations are not necessarily causal; our findings should be treated as indication of an association between poor-quality parenting and cancer rather than a definite proof of it. Further, our study is exploratory and more about establishing associations rather than testing sophisticated mediation models. The use of retrospectively collected data on parenting experiences and childhood SEP makes our study susceptible to measurement bias. Nevertheless, these data are known to have good predictive validity and be inversely associated with morbidity and mortality in ELSA (Demakakos et al. 2012, 2016a, b). Attrition and non-response may have biased to some extent our results (see eTable 5) and likely make our findings a conservative account of the true association between parenting experiences and cancer. In the same vein, survivorship bias might have affected our findings as our study is not inclusive of cancer patients who were diagnosed and died before the age of 55 years. The reliance on self-reported doctor diagnosed cancer is another limitation, which might have led to a misclassification of cancer cases. Further, the collection of cancer data at two-year intervals inevitably introduces length bias that makes our incidence analyses much more relevant to slower progressing types of cancer than aggressive types with life expectancies shorter than 2 years. Finally, we have to acknowledge the reduced statistical power in most of the site-specific analyses, which is a product of the relatively small number of site-specific cancer cases in our data.

Our findings presuppose that childhood experiences of poor parenting are a powerful childhood stressor that can initiate, modulate or catalyse biological processes, which in the long-term and under the right conditions might lead to the development of cancer in late adulthood. The long-term implications of childhood stress-related disregulations for cancer development in adulthood are major, including the promotion of tumorigenesis via different pathways involving oxidative stress and subsequent DNA damage and diminished DNA repair capacity (Antoni et al. 2006; Grivennikov et al. 2010; Jenkins et al. 2014); inflammation (Grivennikov et al. 2010; Hänsel et al. 2010); and activation, replication, and perhaps stimulation of latent oncogenic viruses (Antoni et al. 2006; Green McDonald et al. 2013). Increased reactivity to stress can also influence the tumour microenvironment and facilitate hallmark processes in tumour growth and progression, such as angiogenesis, cell migration, invasion and metastasis and cellular immunosuppression, as well as increased resistance to cancer medications (Antoni et al. 2006; Green McDonald et al. 2013). Further, stress hormones can induce changes in gene expression via modulation of transcription factors (Cole 2013), while childhood experiences of poorer quality parenting may induce epigenetic changes that alter long-term gene expression (Szyf et al. 2008; Champagne and Curley 2009).

In our study, common risk factors, such as unhealthy behaviours and affective problems, do not explain the observed associations and appear to be of little relevance to the underlying pathological processes that were initiated by poor parenting childhood experiences. This finding partially concurs with an earlier proposed model (Kelly-Irving et al. 2013b) as it indicates that parenting problems in childhood may be directly related to cancer risk in adulthood via neuroendocrine and other biological mechanisms, but is discordant with the suggestion that stress-induced unhealthy behaviours may also mediate the observed associations. Nevertheless, our findings on the role of unhealthy behaviours and affective problems should be interpreted with caution mainly for two reasons. First, we did not have data on unhealthy behaviours, psychosocial factors and affective problems from early and middle adulthood, when these factors might be more salient for the examined associations. Second, due to study design limitations, our research did not include many cases of lung cancer and other common cancers known to be strongly related to smoking such as pancreatic, oral cavity, pharyngeal and oesophageal cancers.

Regarding the role of SEP, our findings suggest that the observed associations were neither a product of material disadvantage in childhood nor mediated by adult SEP. Poor-quality parenting seems to be associated with adult cancer risk independent of SEP, but whether SEP moderates this association remains to be examined.

Although all measures of parenting were associated with the risk of cancer in adulthood, it was parental overprotection, and, in particular, paternal overprotection that were more consistently and strongly associated with cancer risk in both men and women. Parental overprotection is a known risk for psychosocial development (Parker 1983). For the importance of paternal overprotection for cancer development, we can only speculate that having an autonomy-restricting overprotective father can be a greater and more damaging stressor than having a mother with the same characteristics, especially when, from an evolutionary perspective, father’s role pertains to granting more autonomy, encouraging independence and preparing the developing child for the challenges of the life outside the family environment (Bögels and Perotti 2011).

Considered together, the associations between childhood experiences of poorer quality parenting and increased cancer prevalence in women and increased cancer incidence in men suggest a potentially earlier onset of all-site cancer in women with poorer quality parenting experiences compared with men with the same experiences. A study of adversity in childhood and cancer development up to the age of 50 years reported concordant findings, with women with adverse childhood experiences having a considerably greater all-site cancer risk than men with the same experiences (Kelly-Irving et al. 2013a). In our data, this important sex difference seems to be mostly driven by the earlier onset of colorectal cancer in women who reported having overprotective parents in childhood, as indicated by the prevalence analyses. Considering that the earlier onset of colorectal cancer in women cannot be explained by sex differences in screening behaviours and oestrogens likely are protective against colorectal cancer, as observed in postmenopausal hormone therapy (Green et al. 2012) and use of oral contraceptives (Luan et al. 2015), we hypothesize that this might be associated with women’s vulnerability to colorectal cancer that is possibly brought about by hormonal imbalance and reduced oestrogen levels, disregulation and disruption of oestrogen-related pathways, and reduced lifetime exposure to oestrogens that may be caused by overprotective parenting and subsequent stress adaptations. Oestradiol is known to be protective against DNA damage and activate oestrogen receptor beta (ERβ), which expression reduces colorectal adenomatous polyps and is reduced in carcinomatous colon tissues compared with normal mucosa (Williams et al. 2016). In our cohort, overprotective parenting and duration of reproductive lifespan, that is the time interval between menarche and menopause during which women can reproduce, are inversely associated (data not shown), and this makes the oestrogen-related hypothesis all the more possible.

The association between poorer quality parenting and breast cancer appears to be complex. Decreasing maternal care was protective against prevalent breast cancer, a finding that is consistent with our hypothesis of the mediating role of a shorter reproductive lifespan and thus reduced lifetime exposure to oestrogens. Given the strong positive association between lifetime exposure to oestrogens and risk of breast cancer (Collaborative Group on Hormonal Factors in Breast Cancer 2012), if poorer quality parenting experiences in childhood led to hormonal imbalance and reduced lifetime exposure to oestrogens, then they might confer a protective effect against breast cancer. We also found a borderline positive association between paternal overprotection and increased risk of incident breast cancer, which was the only association between poorer quality parenting and incident cancer observed in women. This finding likely indicates that pathways other than lifetime exposure to oestrogens may also mediate the association between childhood experiences of poorer quality parenting and breast cancer.

Finally, our findings revealed another intriguing sex difference; all measures of parenting in men were strongly associated with incident skin cancer risk in men, but not in women. The lack of information on the sites of skin cancer makes it impossible to speculate about the implicated pathways. Nevertheless, considering that the complete absence of any association between childhood experiences of poorer quality parenting and skin cancer in women is not a statistical artefact, we can hypothesise that sex differences in skin function (Dao and Kazin 2007) and exposure to sunlight and ultraviolet B irradiation as well as male sex-specific carcinogenic processes (Liu-Smith et al. 2017) are likely implicated in these associations.

Our findings are novel and highlight poor-quality parenting as an independent childhood risk factor for cancer at older ages. They expand on the current understanding of cancer aetiology and add to the debate about the role of psychosocial factors in cancer causation. The universality and importance of parenting from birth to childhood and early adulthood makes our findings pertinent to the vast majority of the population and not just people who experienced social disadvantage or abuse and neglect in childhood. On the understanding that suboptimal parenting style is a potentially modifiable risk factor, our findings can inform prevention strategies and early life interventions. Evidence indicates that parenting prevention programmes may be effective public health interventions (Sanders et al. 2014), yet their prevention potential for adult cancer remains to be proven. Future research should focus on the biological mediators of the observed associations. The dynamic relationships between maternal and paternal parenting and other childhood factors and their role in the observed associations should also be prioritized by future research.

## Electronic supplementary material

Below is the link to the electronic supplementary material.

Supplementary material 1 (DOCX 38 kb)
