# Supplementary material for: Childhood experiences of parenting and cancer risk at older ages: findings from the English Longitudinal Study of Ageing (ELSA)
Source: Int J Public Health. 2018 Jun 14;63(7):823–32. doi: 10.1007/s00038-018-1117-3 (PMC6154018; doi:10.1007/s00038-018-1117-3)
Supplement: Supplementary file 1 — Supplementary material 1 (DOCX 38 kb) [file 38_2018_1117_MOESM1_ESM.docx]

**International Journal of Public Health**

**Childhood experiences of parenting and cancer risk at older ages: Findings from the English Longitudinal Study of Ageing**

**Online Appendix**

**List of eTables**

eTable 1 presents the associations between all-site cancer incidence and prevalence and parenting measures prior to imputing any missing values in any of the parenting measures

eTable 2 presents the descriptive analysis.

eTable 3 presents the associations between all-site cancer incidence and parenting measures in men with additional adjustments for baseline chronic diseases, adverse childhood experiences, and childhood diseases

eTable 4 presents the associations between all-site cancer prevalence and parenting measures in women with additional adjustments for baseline chronic diseases, adverse childhood experiences, and childhood diseases

eTable 5 presents key baseline characteristics according to various categories of non-response. The sample in this analysis consists of everyone who had participated in the second ELSA follow-up interview in 2006-07 (ELSA wave 3) and could had been or was included in our study (n=7,535). This analysis aims to provide basic information about non-response in our study.

| **eTable 1. The Associations Between All-site Cancer Incidence and Prevalence and Parenting Measures by Sex (Without Any Imputations of Missing Values in the Parenting Measures), English Longitudinal Study of Ageing, 2007-2013** | | | | |
| --- | --- | --- | --- | --- |
|  | **Cancer Incidence** | | **Cancer Prevalence** | |
|  | **Men** | **Women** | **Men** | **Women** |
| ***No. of participants*** | *1583* | *1838* | *1893* | *2193* |
| ***No. of cases*** | *109* | *78* | *178* | *240* |
| ***Person years of follow-up*** | *7421* | *8812* | *n/a* | *n/a* |
|  | **Hazard ratio (95% CI)^†^** | | **Odds ratio (95% CI)^‡^** | |
| **Parenting Summary score (both parents) – reversed (range: 0-highest quality parenting to 42-lowest quality parenting)** |  |  |  |  |
| Model 1^§^ | 1.05 (1.02 to 1.09) | 1.00 (0.96 to 1.04) | 0.99 (0.96 to 1.02) | 1.02 (0.99 to 1.04) |
| Model 2^¶^ | 1.06 (1.02 to 1.09) | 1.00 (0.96 to 1.04) | 1.00 (0.97 to 1.03) | 1.02 (1.00 to 1.04) |
| Model 3^#^ | 1.06 (1.02 to 1.09) | 1.00 (0.96 to 1.04) | 1.00 (0.97 to 1.03) | 1.02 (1.00 to 1.04) |
| Model 4^ǁ^ | 1.05 (1.01 to 1.09) | 1.00 (0.96 to 1.04) | 1.00 (0.97 to 1.03) | 1.02 (1.00 to 1.04) |
| **Maternal Care score – reversed (range: 0-highest levels of care to 9-lowest levels of care)** |  |  |  |  |
| Model 1^§^ | 1.12 (1.01 to 1.24) | 0.99 (0.89 to 1.11) | 0.97 (0.88 to 1.07) | 1.00 (0.93 to 1.07) |
| Model 2^¶^ | 1.14 (1.02 to 1.26) | 1.00 (0.89 to 1.12) | 1.00 (0.90 to 1.10) | 1.00 (0.94 to 1.07) |
| Model 3^#^ | 1.14 (1.02 to 1.27) | 0.99 (0.88 to 1.11) | 1.00 (0.90 to 1.11) | 1.01 (0.94 to 1.08) |
| Model 4^ǁ^ | 1.12 (1.00 to 1.25) | 1.00 (0.89 to 1.13) | 0.99 (0.90 to 1.10) | 1.00 (0.93 to 1.08) |
| **Maternal Overprotection score (range: 0-lowest levels of overprotection to 12-highest levels of overprotection)** |  |  |  |  |
| Model 1^§^ | 1.11 (1.01 to 1.22) | 0.98 (0.87 to 1.09) | 0.98 (0.90 to 1.06) | 1.07 (1.00 to 1.14) |
| Model 2^¶^ | 1.12 (1.02 to 1.23) | 0.98 (0.87 to 1.10) | 0.99 (0.91 to 1.07) | 1.07 (1.00 to 1.14) |
| Model 3^#^ | 1.12 (1.02 to 1.24) | 0.97 (0.87 to 1.04) | 0.98 (0.90 to 1.07) | 1.07 (1.01 to 1.15) |
| Model 4^ǁ^ | 1.10 (0.99 to 1.22) | 0.97 (0.86 to 1.10) | 0.99 (0.90 to 1.08) | 1.07 (1.00 to 1.15) |
| **Paternal Care score – reversed (range: 0-highest levels of care to 9-lowest levels of care)** |  |  |  |  |
| Model 1^§^ | 1.13 (1.03 to 1.24) | 1.03 (0.92 to 1.16) | 0.94 (0.86 to 1.02) | 1.01 (0.94 to 1.09) |
| Model 2^¶^ | 1.15 (1.05 to 1.26) | 1.04 (0.92 to 1.17) | 0.97 (0.89 to 1.06) | 1.02 (0.95 to 1.10) |
| Model 3^#^ | 1.15 (1.05 to 1.26) | 1.03 (0.92 to 1.17) | 0.97 (0.89 to 1.06) | 1.02 (0.95 to 1.10) |
| Model 4^ǁ^ | 1.13 (1.02 to 1.25) | 1.04 (0.92 to 1.18) | 0.97 (0.88 to 1.06) | 1.01 (0.94 to 1.09) |
| **Paternal Overprotection score (range: 0-lowest levels of overprotection to 12-highest levels of overprotection)** |  |  |  |  |
| Model 1^§^ | 1.15 (1.04 to 1.28) | 0.99 (0.88 to 1.11) | 1.00 (0.91 to 1.09) | 1.08 (1.01 to 1.16) |
| Model 2^¶^ | 1.16 (1.05 to 1.29) | 0.99 (0.88 to 1.11) | 1.01 (0.92 to 1.10) | 1.09 (1.02 to 1.16) |
| Model 3^#^ | 1.17 (1.05 to 1.29) | 0.98 (0.88 to 1.11) | 1.01 (0.92 to 1.10) | 1.09 (1.02 to 1.17) |
| Model 4^ǁ^ | 1.14 (1.02 to 1.27) | 0.98 (0.87 to 1.11) | 1.02 (0.93 to 1.12) | 1.09 (1.02 to 1.17) |
| ^†^Hazard ratios denote hazard change per unit change in the predictor  ^‡^Odds ratios denote change in the odds per unit change in the predictor  ^§^This is the unadjusted association | | | | |
| ^¶^Model 2 is adjusted for age | | | | |
| ^#^As model 2, plus adjustment for childhood socioeconomic position (i.e. ownership of the first ever permanent residence, number of books in the household at age 10 years, and father’s or main carer’s occupational class at age 14 years) | | | | |
| ^ǁ^As model 3, plus adjustment for adult socioeconomic position (i.e. education and total net household wealth), marital status, parenthood status, obesity (i.e. body mass index and waist circumference), memory, unhealthy behaviours (i.e. smoking and physical activity), social factors (i.e. social support and number of problems with social relationships), elevated depressive symptoms, and positive affect | | | | |

| **eTable 2**. **The baseline characteristics of the all-site cancer incidence sample by sex, English Longitudinal Study of Ageing, 2007** | | | | | | |
| --- | --- | --- | --- | --- | --- | --- |
|  | **Men** | | | **Women** | | |
|  | **N (%)^*^** | **Mean parenting summary score**^§^ **(SD)** | ***P value*^†^** | **N (%)^*^** | **Mean parenting summary score**^§^ **(SD)** | ***P value*^†^** |
| **No. of participants** | 1650 |  |  | 1984 |  |  |
| **Mean age, years, (SD)** | 67.3 (8.5) | 11.4 (5.5) |  | 67.9 (9.0) | 11.9 (6.0) |  |
| **Marital status (%)** |  |  | 0.43 |  |  | 0.42 |
| Married | 1342 (81.3) | 11.4 (5.5) |  | 1252 (63.1) | 11.8 (6.1) |  |
| Other | 308 (18.7) | 11.1 (5.7) |  | 732 (36.9) | 12.0 (5.9) |  |
| **Childless (without any children)** |  |  | 0.19 |  |  | 0.098 |
| No | 1473 (89.3) | 11.3 (5.5) |  | 1764 (88.9) | 12.0 (6.1) |  |
| Yes | 177 (10.7) | 11.9 (5.6) |  | 220 (11.1) | 11.3 (5.3) |  |
| **First ever residence was rented** |  |  | 0.82 |  |  | 0.91 |
| No | 470 (28.5) | 11.4 (5.8) |  | 502 (25.3) | 11.8 (6.0) |  |
| Yes | 1118 (67.8) | 11.3 (5.3) |  | 1401 (70.6) | 11.9 (6.1) |  |
| Missing^‡^ | 62 (3.8) | 11.6 (5.5) |  | 81 (4.1) | 12.7 (5.1) |  |
| **No. of books at the household at age 10 years** |  |  | 0.007 |  |  | <0.001 |
| None or very few (0-10 books) | 459 (27.8) | 11.9 (5.7) |  | 442 (22.3) | 13.3 (6.2) |  |
| Enough to fill one shelf (11-25 books) | 392 (23.8) | 11.4 (5.2) |  | 489 (24.6) | 11.7 (5.9) |  |
| Enough to fill one bookcase (26-100 books) | 492 (29.9) | 11.3 (5.4) |  | 595 (30.0) | 11.5 (5.9) |  |
| Enough to fill two bookcases (≥101 books) | 230 (13.9) | 10.4 (5.8) |  | 381 (19.2) | 11.2 (6.0) |  |
| Missing^‡^ | 76 (4.6) | 11.3 (5.3) |  | 77 (3.9) | 12.1 (6.2) |  |
| **Paternal occupational class at age 14 years (%)** |  |  | 0.94 |  |  | 0.95 |
| Managerial and professional occupations / business owner | 523 (31.7) | 11.4 (5.6) |  | 692 (34.9) | 11.8 (6.0) |  |
| Intermediate occupations | 577 (35.0) | 11.2 (5.4) |  | 592 (29.8) | 11.9 (6.3) |  |
| Routine occupations / casual jobs / unemployed / disabled | 509 (30.8) | 11.3 (5.5) |  | 619 (31.2) | 11.9 (5.8) |  |
| Other (including 11 cases of missing values)^‡^ | 41 (2.5) | 13.1 (6.1) |  | 81 (4.1) | 12.3 (6.8) |  |
| **Education (%)** |  |  | 0.23 |  |  | 0.59 |
| A-level or higher | 855 (51.8) | 11.6 (5.5) |  | 629 (31.7) | 12.0 (6.3) |  |
| GCSE / O-level / other qualification | 449 (27.2) | 11.1 (5.6) |  | 705 (35.5) | 11.7 (6.2) |  |
| No educational qualifications | 346 (21.0) | 11.2 (5.3) |  | 650 (32.8) | 12.0 (5.6) |  |
| **Total net household wealth tertiles, pound sterling, (%)** |  |  | 0.27 |  |  | 0.39 |
| Highest (≥£306,100) | 665 (40.3) | 11.1 (5.2) |  | 663 (33.4) | 11.7 (6.0) |  |
| Intermediate (<£306,100 to ≥£151,540) | 564 (34.2) | 11.4 (5.6) |  | 700 (35.3) | 11.8 (5.8) |  |
| Lowest (<£151,540) | 421 (25.5) | 11.7 (5.8) |  | 621 (31.3) | 12.2 (6.4) |  |
| **Smoking (%)** |  |  | 0.81 |  |  | <0.001 |
| Never smoker | 484 (29.3) | 11.3 (5.5) |  | 919 (46.3) | 11.5 (5.9) |  |
| Former smoker | 974 (59.0) | 11.4 (5.4) |  | 830 (41.8) | 12.5 (6.1) |  |
| Current smoker | 192 (11.7) | 11.6 (5.8) |  | 235 (11.9) | 11.5 (6.4) |  |
| **Physical activity at least once a week (%)** |  |  | 0.80 |  |  | 0.051 |
| Physically inactive | 106 (6.4) | 11.3 (5.7) |  | 116 (5.8) | 12.8 (5.7) |  |
| Mild-intensity | 143 (8.7) | 11.3 (5.8) |  | 345 (17.4) | 12.5 (6.2) |  |
| Moderate-intensity | 845 (51.2) | 11.3 (5.4) |  | 1006 (50.7) | 11.8 (6.1) |  |
| Vigorous-intensity | 556 (33.7) | 11.5 (5.5) |  | 517 (26.1) | 11.5 (5.8) |  |
| **Body mass index (%)** |  |  | 0.38 |  |  | 0.055 |
| <25kg/m^2^ | 349 (21.1) | 11.6 (5.6) |  | 534 (26.9) | 11.6 (6.1) |  |
| 25 to <30 kg/m^2^ | 750 (45.5) | 11.2 (5.4) |  | 665 (33.5) | 11.7 (5.8) |  |
| ≥ 30 kg/m2 | 379 (23.0) | 11.6 (5.7) |  | 542 (27.3) | 12.4 (6.1) |  |
| Missing^‡^ | 172 (10.4) | 11.2 (5.1) |  | 243 (12.3) | 12.2 (6.4) |  |
| **Waist Circumference (%)** |  |  | 0.80 |  |  | 0.99 |
| <94cm (in men) / <80cm (in women) | 392 (23.8) | 11.5 (5.8) |  | 363 (18.3) | 11.9 (6.4) |  |
| ≥94 to 101cm (in men) / ≥80 to 87cm (in women) | 423 (25.7) | 11.3 (5.0) |  | 414 (20.9) | 11.9 (5.9) |  |
| ≥102cm (in men) / ≥88cm (in women) | 691 (41.8) | 11.4 (5.8) |  | 994 (50.1) | 11.9 (5.9) |  |
| Missing^‡^ | 144 (8.7) | 10.9 (4.6) |  | 213 (10.7) | 11.9 (6.4) |  |
| **Elevated depressive symptoms (%)** |  |  | 0.003 |  |  | <0.001 |
| No | 1509 (91.4) | 11.2 (5.4) |  | 1681 (84.7) | 11.6 (5.9) |  |
| Yes | 141 (8.6) | 12.7 (6.3) |  | 303 (15.3) | 13.6 (6.5) |  |
| **Memory (recall word summary score) (range:0-20)** |  |  | 0.72 |  |  | 0.28 |
| Highest tertile (≥12 recalled words) | 624 (37.8) | 11.4 (5.5) |  | 841 (42.4) | 12.1 (6.2) |  |
| Intermediate tertile (11 to 9 recalled words) | 565 (34.3) | 11.4 (5.6) |  | 657 (33.1) | 11.6 (5.9) |  |
| Lowest tertile (≤8 recalled words) | 461 (27.9) | 11.2 (5.4) |  | 486 (24.5) | 12.1 (5.9) |  |
| **Social Support** |  |  | <0.001 |  |  | <0.001 |
| High levels | 1007 (61.0) | 10.6 (5.4) |  | 1237 (62.3) | 11.2 (6.0) |  |
| Low levels | 552 (33.5) | 12.4 (5.5) |  | 638 (32.2) | 13.1 (5.7) |  |
| Missing^‡^ | 91 (5.5) | 12.9 (5.4) |  | 109 (5.5) | 12.6 (6.9) |  |
| **Problems with social relationships** |  |  | <0.001 |  |  | <0.001 |
| No | 956 (58.0) | 10.6 (5.3) |  | 1139 (57.4) | 11.3 (5.8) |  |
| Yes - with one type of social relationships | 380 (23.0) | 12.0 (5.4) |  | 495 (25.0) | 12.5 (6.2) |  |
| Yes - with two or more types of social relationships | 218 (13.2) | 13.1 (5.9) |  | 236 (11.9) | 13.4 (6.3) |  |
| Missing^‡^ | 96 (5.8) | 13.0 (5.6) |  | 114 (5.7) | 12.6 (6.8) |  |
| **Positive affect score (range:0-15)** |  |  | <0.001 |  |  | <0.001 |
| Highest tertile (score: 15) | 591 (35.8) | 10.0 (5.3) |  | 780 (39.3) | 10.6 (5.8) |  |
| Intermediate tertile (score: ≤14 to ≥13) | 499 (30.3) | 11.3 (5.1) |  | 587 (29.6) | 12.3 (5.9) |  |
| Lowest tertile (score: ≤12) | 439 (26.6) | 13.1 (5.6) |  | 466 (23.5) | 13.2 (6.1) |  |
| Missing^‡^ | 121 (7.3) | 12.0 (5.7) |  | 151 (7.6) | 12.6 (6.6) |  |
| ^*^Denotes the number of participants in each category (with the respective percent in brackets) unless otherwise stated | | | | | | |
| ^†^*P values* were generated using the analysis of variance test | | | | | | |
| ^‡^The category of missing / other was not used in the calculation of the *P value*  ^§^The parenting summary score is reversed and ranges from 0 (highest quality parenting) to 42 (poorest quality parenting) | | | | | | |

| **eTable 3. The Associations Between All-site Cancer Incidence and Parenting Measures with Additional Adjustments in Men, English Longitudinal Study of Ageing, 2007-2013^†^** | |
| --- | --- |
| ***No. of participants*** | *1650* |
| ***No. of cases*** | *112* |
| ***Person years of follow-up*** | *7712* |
| **Parenting Summary score (both parents) – reversed (range: 0-highest quality parenting to 42-lowest quality parenting)** |  |
| Model 1 HR (95% CI)^§^ | 1.04 (1.01 to 1.08) |
| Model 2 HR (95% CI)^¶^ | 1.05 (1.01 to 1.09) |
| Model 3 HR (95% CI)^#^ | 1.04 (1.01 to 1.08) |
| **Maternal Care score – reversed (range: 0-highest levels of care to 9-lowest levels of care)** |  |
| Model 1 HR (95% CI)^§^ | 1.10 (0.99 to 1.23) |
| Model 2 HR (95% CI)^¶^ | 1.11 (0.99 to 1.23) |
| Model 3 HR (95% CI)^#^ | 1.09 (0.97 to 1.21) |
| **Maternal Overprotection score (range: 0-lowest levels of overprotection to 12-highest levels of overprotection)** |  |
| Model 1 HR (95% CI)^§^ | 1.07 (0.97 to 1.19) |
| Model 2 HR (95% CI)^¶^ | 1.08 (0.98 to 1.19) |
| Model 3 HR (95% CI)^#^ | 1.06 (0.96 to 1.18) |
| **Paternal Care score – reversed (range: 0-highest levels of care to 9-lowest levels of care)** |  |
| Model 1 HR (95% CI)^§^ | 1.12 (1.01 to 1.23) |
| Model 2 HR (95% CI)^¶^ | 1.14 (1.04 to 1.26) |
| Model 3 HR (95% CI)^#^ | 1.12 (1.01 to 1.24) |
| **Paternal Overprotection score (range: 0-lowest levels of overprotection to 12-highest levels of overprotection)** |  |
| Model 1 HR (95% CI)^§^ | 1.13 (1.02 to 1.26) |
| Model 2 HR (95% CI)^¶^ | 1.15 (1.03 to 1.28) |
| Model 3 HR (95% CI)^#^ | 1.13 (1.01 to 1.26) |
| ^†^Hazard ratios denote hazard change per unit change in the predictor  ^§^Model 1 is estimated for a sample of 1649 male participants (no. of cases=112) and is adjusted for age, childhood socioeconomic position (i.e. ownership of the first ever permanent residence, number of books in the household at age 10 years, and father’s or main carer’s occupational class at age 14 years), adult socioeconomic position (i.e. education and total net household wealth), marital status, parenthood status, obesity (i.e. body mass index and waist circumference), cognitive function, unhealthy behaviours (i.e. smoking and physical activity), social factors (i.e. social support and number of problems with social relationships), elevated depressive symptoms, positive affect and self-reported doctor diagnosed chronic diseases at baseline (i.e. heart disease, stroke, psychiatric problems, and chronic lung disease) | |
| ^¶^Model 2 is adjusted for age, childhood socioeconomic position (i.e. ownership of the first ever permanent residence, number of books in the household at age 10 years, and father’s or main carer’s occupational class at age 14 years), adult socioeconomic position (i.e. education and total net household wealth), marital status, parenthood status, obesity (i.e. body mass index and waist circumference), cognitive function, unhealthy behaviours (i.e. smoking and physical activity), social factors (i.e. social support and number of problems with social relationships), elevated depressive symptoms, positive affect, and self-reported childhood health problems (i.e. epilepsy, asthma, diabetes, hearing problems, limiting disability, and emotional, nervous, or psychiatric problems at age <16 years) | |
| ^#^Model 3 is adjusted for age, childhood socioeconomic position (i.e. ownership of the first ever permanent residence, number of books in the household at age 10 years, and father’s or main carer’s occupational class at age 14 years), adult socioeconomic position (i.e. education and total net household wealth), marital status, parenthood status, obesity (i.e. body mass index and waist circumference), cognitive function, unhealthy behaviours (i.e. smoking and physical activity), social factors (i.e. social support and number of problems with social relationships), elevated depressive symptoms, positive affect and self-reported adverse childhood experiences (i.e. physically abusive parents; parental mental health or addiction problems; or experience of physical or sexual assault at age <16 years) | |

| **eTable 4. The Associations Between All-site Cancer Prevalence and Parenting Measures with Additional Adjustments in Women, English Longitudinal Study of Ageing, 2007^†^** | |
| --- | --- |
| ***No. of participants*** | *2375* |
| ***No. of cases*** | *262* |
| **Parenting Summary score (both parents) – reversed (range: 0-best parenting to 42-worst parenting)** |  |
| Model 1 OR (95% CI) ^§^ | 1.02 (1.00 to 1.04) |
| Model 2 OR (95% CI)^¶^ | 1.02 (1.00 to 1.04) |
| Model 3 OR (95% CI)^#^ | 1.02 (1.00 to 1.04) |
| **Maternal Care score – reversed (range: 0-highest levels of care to 9-lowest levels of care)** |  |
| Model 1 OR (95% CI) ^§^ | 1.01 (0.94 to 1.08) |
| Model 2 OR (95% CI)^¶^ | 1.01 (0.94 to 1.08) |
| Model 3 OR (95% CI)^#^ | 1.00 (0.93 to 1.07) |
| **Maternal Overprotection score (range: 0-lowest levels of overprotection to 12-highest levels of overprotection)** |  |
| Model 1 OR (95% CI) ^§^ | 1.06 (1.00 to 1.14) |
| Model 2 OR (95% CI)^¶^ | 1.06 (1.00 to 1.13) |
| Model 3 OR (95% CI)^#^ | 1.06 (0.99 to 1.13) |
| **Paternal Care score – reversed (range: 0-highest levels of care to 9-lowest levels of care)** |  |
| Model 1 OR (95% CI) ^§^ | 1.03 (0.96 to 1.11) |
| Model 2 OR (95% CI)^¶^ | 1.03 (0.96 to 1.11) |
| Model 3 OR (95% CI)^#^ | 1.02 (0.95 to 1.10) |
| **Paternal Overprotection score (range: 0-lowest levels of overprotection to 12-highest levels of overprotection)** |  |
| Model 1 OR (95% CI) ^§^ | 1.09 (1.02 to 1.17) |
| Model 2 OR (95% CI)^¶^ | 1.09 (1.02 to 1.17) |
| Model 3 OR (95% CI)^#^ | 1.09 (1.02 to 1.16) |
| ^†^Odds ratios denote change in the odds per unit change in the predictor  ^§^Model 1 is estimated for a sample of 2374 participants (no. of cases=262) and is adjusted for age, childhood socioeconomic position (i.e. ownership of the first ever permanent residence, number of books in the household at age 10 years, and father’s or main carer’s occupational class at age 14 years), adult socioeconomic position (i.e. education and total net household wealth), marital status, parenthood status, obesity (i.e. body mass index and waist circumference), cognitive function, unhealthy behaviours (i.e. smoking and physical activity), social factors (i.e. social support and number of problems with social relationships), elevated depressive symptoms, positive affect and self-reported doctor diagnosed chronic diseases at baseline (i.e. heart disease, stroke, psychiatric problems, and chronic lung disease) | |
| ^¶^Model 2 is adjusted for age, childhood socioeconomic position (i.e. ownership of the first ever permanent residence, number of books in the household at age 10 years, and father’s or main carer’s occupational class at age 14 years), adult socioeconomic position (i.e. education and total net household wealth), marital status, parenthood status, obesity (i.e. body mass index and waist circumference), cognitive function, unhealthy behaviours (i.e. smoking and physical activity), social factors (i.e. social support and number of problems with social relationships), elevated depressive symptoms, positive affect, and self-reported childhood health problems (i.e. epilepsy, asthma, diabetes, hearing problems, limiting disability, and emotional, nervous, or psychiatric problems at age <16 years) | |
| ^#^Model 3 is adjusted for age, childhood socioeconomic position (i.e. ownership of the first ever permanent residence, number of books in the household at age 10 years, and father’s or main carer’s occupational class at age 14 years), adult socioeconomic position (i.e. education and total net household wealth), marital status, parenthood status, obesity (i.e. body mass index and waist circumference), cognitive function, unhealthy behaviours (i.e. smoking and physical activity), social factors (i.e. social support and number of problems with social relationships), elevated depressive symptoms, positive affect and self-reported adverse childhood experiences (i.e. physically abusive parents; parental mental health or addiction problems; or experience of physical or sexual assault at age <16 years) | |

| **eTable 5. Key Baseline Characteristics According to Participation in Our Study, English Longitudinal Study of Ageing, 2007** | | | | | | |
| --- | --- | --- | --- | --- | --- | --- |
|  | **Participated in the 2^nd^ ELSA follow-up interview (ELSA wave 3) in 2006-07, but not in the life history Interview in 2007**  **(n=1336)** | **Non-response to the parenting questions or were not reared by both natural parents (n=797)** | **Non-response to the childhood experiences questionnaire (n=931)** | **Excluded because of missing values in the analysis variables (n=110)** | **Included in the study**  **(n=4361)** | ***P Value*** |
| Mean age (SD) | 66.3 (10.9) | 65.77 (9.5) | 63.5 (10.7) | 61.9 (9.8) | 63.4 (8.9) | <0.001 |
| Male | 597 (44.7) | 310 (38.9) | 419 (45.0) | 42 (38.2) | 1986 (45.5) | 0.008 |
| Married | 822 (61.5) | 473 (59.3) | 494 (53.1) | 93 (84.6) | 3083 (70.1) | <0.001 |
| No educational qualifications | 574 (43.0) | 344 (43.2) | 313 (33.6) | 26 (23.6) | 1226 (28.1) | <0.001 |
| Lowest total net household wealth tertile | 560 (45.0) | 299 (37.8) | 386 (42.5) | 13 (46.6) | 1187 (27.2) | <0.001 |
| Elevated depressive symptoms (≥4 symptoms) | 228 (20.2) | 146 (18.5) | 155 (16.9) | 9 (10.8) | 544 (12.5) | <0.001 |
